# Steps toward more complete reporting of systematic reviews of diagnostic test accuracy: Preferred Reporting Items for Systematic Reviews and Meta-Analyses of Diagnostic Test Accuracy (PRISMA-DTA)

**DOI:** 10.1186/s13643-019-1090-9

**Published:** 2019-07-11

**Authors:** Trevor A. McGrath, David Moher, Matthew D. F. McInnes

**Affiliations:** 1 Department of Radiology, University of Ottawa, Ottawa, Canada; 2 Centre for Journalology, Clinical Epidemiology Program, The Ottawa Hospital Research Institute, Ottawa, Canada; 3 Clinical Epidemiology Program, Department of Radiology, The Ottawa Hospital Research Institute, University of Ottawa, Room c159 Ottawa Hospital Civic Campus, 1053 Carling Ave, Ottawa, Ontario K1Y 4E9 Canada

**Keywords:** Systematic reviews, Diagnostic test accuracy, PRISMA-DTA, Completeness of reporting

## Abstract

Reporting standards in biomedical research have been shown to be suboptimal. The publication of the PRISMA statement has improved the completeness of reporting of systematic reviews, but several issues specific to diagnostic test accuracy are not included in the PRISMA statement. Therefore, a diagnostic test accuracy extension of the PRISMA statement, PRISMA-DTA, was created. This commentary addresses completeness of reporting in systematic reviews, the PRISMA-DTA statement, and strategies for optimal uptake of reporting guidelines.

## Background

Inadequate reporting in biomedical research is a long-recognized issue, with the concern being that it does not allow for assessment of methodological quality and applicability of results, and consequently leads to wasted research that may be prone to bias [1, 2]. Poorly reported randomized controlled trials are associated with exaggerated treatment effects [3]. The EQUATOR Network was launched in 2008 to facilitate the development and dissemination of reporting guidelines to improve the quality of reporting in biomedical literature [4]. The Preferred Reporting Items for Systematic Reviews and Meta-Analyses (PRISMA) statement was published in 2009 with the goal to improve reporting of systematic reviews and meta-analysis and reducing research waste [2, 5, 6]. Since the publication of the PRISMA statement, reporting of systematic reviews has improved in several areas [7, 8].

While the focus of PRISMA was to help improve the completeness of reporting systematic reviews examining the effects of health interventions, several key issues in systematic reviews of diagnostic test accuracy (DTA) were not adequately addressed [9–12]. Several important differences exist between the conduct of a systematic review of interventions compared to a review of DTA. These differences also need to be transparently reported. Systematic reviews of interventions, and the PRISMA statement, reference the clinical question in terms of Participants, Intervention, Comparison, and Outcomes (PICO) whereas in DTA systematic reviews, Participants, Index test(s), and Target condition (PIT) is more applicable [5, 9]. Data handling also differs between DTA systematic reviews versus systematic reviews of interventions, with issues such as multiple thresholds for test positivity, multiple readers of the index test, and indeterminate test results [9]. Additionally, since sensitivity and specificity are correlated and vary with respect to the diagnostic threshold, hierarchical methods are generally recommended for meta-analysis of DTA [12, 13]. Using hierarchical pooling methods has been shown to give significantly different results than non-hierarchical methods and thus reporting of the methods used for meta-analysis is critically important in DTA systematic reviews. To address these unique issues, and others, an extension to PRISMA for authors reporting reviews of DTA called PRISMA-DTA was developed. The onus to improve the reporting of DTA systematic reviews falls on everybody involved in the publication of a manuscript, from the study authors, to journal peer reviewers and journal editors; and thus, awareness of PRISMA-DTA is of importance to anyone involved in the DTA systematic review research domain.

## PRISMA-DTA

A systematic review of the literature regarding reporting of DTA systematic reviews was performed followed by a three-round Delphi process and an in-person consensus meeting to generate the 27-item Preferred Reporting Items for Systematic Reviews and Meta-Analyses of Diagnostic Test Accuracy (PRISMA-DTA) extension of the original PRISMA statement [9, 10]. PRISMA-DTA for abstracts was also developed. The *PRISMA-DTA and PRISMA-DTA for abstracts* publication is available (open-access) in the *Journal of the American Medical Association* (JAMA) [9]. PRISMA-DTA and PRISMA-DTA for abstracts user-friendly checklists for authors and reviewers are available in both Word and PDF formats from both *The EQUATOR Network* and the *PRISMA* websites [5, 14]. This extension of the PRISMA statement should be used for all DTA systematic reviews performed moving forward. Additionally, submissions of DTA systematic reviews to conferences should be adherent to PRISMA-DTA for abstracts as conference abstracts are often published and, like published manuscripts, should meet minimum reporting standards.

## Reporting guideline uptake

Optimal uptake of reporting guidelines has three main constituents: endorsement, implementation, and adherence. Endorsement is a policy decision by a journal to recommend that authors submit the appropriate reporting guideline for their research type, as is the case with the journal *Systematic Reviews*, along with other leading journals in fields commonly producing DTA systematic reviews, such as *Clinical Chemistry* and *Radiology.* Implementation is the action(s) taken by journals to ensure that authors adhere to an endorsed reporting guideline. This may include requiring a checklist of the relevant reporting guideline be submitted with the manuscript prior to consideration of the manuscript by the journal editorial team. Adherence refers to action(s) taken by authors to ensure that a research report is compliant with the items recommended by the appropriate/relevant reporting guideline. Adherence is the responsibility of the manuscript author to create well-reported and reproducible research. As the editorial burden of manually assessing and enforcing adherence of all submitted manuscripts would be enormous, enforcement of adherence currently largely relies on peer reviewers and editors. From the perspective of the author submitting a review, using PRISMA-DTA will ideally improve the completeness and clarity of reporting of the submitted work, perhaps aiding peer reviewers and editors to more quickly reach a decision of the merits of the review for publication.

## Solutions

Reporting of systematic reviews has been shown to be improved since the publication of the PRISMA statement [8]. Studies have shown that journal endorsement of reporting guidelines does not necessarily improve the completeness of reporting of systematic reviews [15, 16]. However, in a recent assessment of the adherence of DTA systematic reviews to PRISMA-DTA, it was found that reviews citing PRISMA or an extension of PRISMA had significantly more complete reporting than reviews that did not [16]. The lack of improvement in reporting seen in journals endorsing reporting guidelines versus journals that did not is potentially confounded by authors using reporting guidelines despite lack of journal endorsement. In the 100 reviews in the PRISMA-DTA reporting assessment, two thirds cited PRISMA or a PRISMA extension, whereas only one third were submitted to journals endorsing reporting guidelines for systematic reviews [16]. Journal endorsement of reporting guidelines, although not shown in isolation to improve DTA systematic review reporting, can help drive the overall use of reporting guidelines, which was shown to improve reporting of DTA systematic reviews [16]. Thus, we encourage all journals to endorse reporting guidelines.

While fixed abstract word count limitations by journals were not associated with completeness of abstract reporting, it was shown that abstracts with higher word counts were reported more completely [16]. Thus, we encourage authors, if faced with a fixed word count limit for the abstract, to be as complete as possible and use the word count allotment fully. Additionally, studies using supplemental material were reported more completely [16]. If authors are faced with full-text word count limits, restrictions on figures and tables, or other constraints, we encourage the use of online supplemental material which can be mentioned in the full text of the article to steer readers towards complete information for their review. As motivation for review authors, if required, it was shown that systematic reviews published in high impact radiology journals were cited more often when reporting was more complete [17].

Several DTA-specific issues were found to be reported in less than one third of reviews in the PRISMA-DTA adherence assessment [16]. These include the clinical setting, which has been shown to influence test accuracy, as well as definitions of the target condition, index test, and reference standard [16]. Systematic reviews of DTA pose unique methodological and thus reporting issues not seen in systematic reviews of interventions. The originally published PRISMA statement was not designed to address these issues, and thus, a specialized DTA reporting guideline should be used to ensure adequate DTA-specific reporting for all DTA systematic reviews moving forward.

Interestingly, a recently published study found that study authors found reporting guidelines to be most useful when used early in the research process, rather than after the manuscript was complete or requested by a journal editor [18]. This shows that the perceived value of reporting guidelines is highest during the planning and protocol development phase. Knowing this, it seems an emphasis with respect to reporting guidelines should be made when educating biomedical researchers about different study designs. If researchers are aware of reporting guidelines and can use them early in the research process, when they have shown to be perceived as the most helpful, then hopefully usage rates of reporting guidelines on the whole will improve.

## Conclusion

With the publication of the PRISMA-DTA and PRISMA-DTA for abstract checklists and the continued efforts of authors, peer reviewers, and journal editors, hopefully we can raise the quality of reporting in DTA systematic reviews.

## Data Availability

Not applicable

## Abbreviations

DTA: Diagnostic test accuracy
JAMA: Journal of the American Medical Association
PICO: Participants, Intervention, Comparison, and Outcomes
PIT: Participants, Index test(s), and Target condition
PRISMA: Preferred Reporting Items for Systematic Reviews and Meta-Analyses
PRISMA-DTA: Preferred Reporting Items for Systematic Reviews and Meta-Analyses for Diagnostic test accuracy
